# The Treatment of Chorea

**Published:** 1908-06

**Authors:** Arthur Francis Voelcker

**Affiliations:** Physician to the Hospital for Sick Children, Great Ormond Street. London, Physician to Out-patients, Middlesex Hospital


### The Treatment of Chorea.

By ARTHUR FRANCIS VOELCKER, M. D., F.R.C.P.
Physician to the Hospital for Sick Children, Great Ormond Street, London, Physician to Out-patients, Middlesex Hospital.

[*Folia Therapeutica, April, 1908.*]

AMONG the curable diseases of the nervous system there are few in the management of which more varied modes of treatment have been adopted than in chorea. This is due partly to the various forms in which the disease shows itself and partly to the views which are held as to the nature of the disease; thus we see one line of treatment which is directed to the quieting of the motor, another to the mental phenomena, and a third to the combating of the rheumatic processes which are held by so many to be at the root of the choreic manifestations.

There are those who hold that drug treatment is useless—that the disease will run its course and that it is uninfluenced by drug treatment—while others have seen very definite and beneficial results following on the use of drugs. My own experience has taught me that a very distinct improvement in our treatment of those forms of chorea which are characterised by excessive movement or mental upset has taken place in the last few years owing to the introduction of some of the newer sedative agents. Chorea, like any other morbid manifestation, is not to be treated by drugs alone. The management of a case of chorea calls for a considerable amount of tact as well as of therapeutic skill on the part of the physician. Drugs administered without due regard to the surroundings, diet and general hygiene of the patient are worse than useless, and it is a well-known fact that many cases of chorea recover without any drug treatment whatever.

In the following contribution I propose to set out the general lines which we should follow in the treatment of a case of chorea, and to indicate what are the special phenomena which may arise and the treatment appropriate for them.

We may divide the treatment of chorea into three heads: (1) general; (2) symptomatic; and (3) specific.

(1) *General.*—Directly a case of chorea is recognised the child should be taken from school, or if at home should be kept apart from the other children in the house, at all events for the greater part of the day. So often the early stages of the disease go unrecognised because it is not appreciated that the little outbursts of temper, irritability, spitefulness, peevishness, or emotional instability are the early evidences of the condition, even before there is any definite motor evidence of the disease. The recognition of this fact will often prevent the outbreak of a violent manifestation of choreic movements which may follow on the injudicious, though well-intentioned, correction of faults which are themselves only the result of a disease in its earliest stages. When we remember the natural fidgetiness of children and the imperceptible line which divides a natural from a pathological restlessness, we can see how easy it is, if we rely merely on motor phenomena, to overlook or misinterpret the early stages of the disease. It is on this account that leniency must be shown to the shortcomings of children, and particular care taken that the children are as far as possible kept away from those environments which have in them the makings of a difference of opinion or of rivalry of interest. At this stage much harm may be done by injudicious parents, foolish nurses, or teasing brothers or sisters. In many of these cases, and especially if the child has already had one attack of chorea, an attack may be aborted by taking the child out of its usual surroundings and sending it away into quieter ones with a relative or nurse who is well known and congenial to the patient. To send cases at this stage to strangers or to convalescent homes is worse than useless. Isolation in the early stages is undesirable, if by isolation we mean shutting a child up by itself. Rest is a very important requirement at this stage, but note must be taken of the temperament of the child, for in some cases the forcible detention of a child in bed all day long will do more harm than good. On the other hand, it is essential that the child should be made to lie down for at least part of the day, though there is no reason why this procedure should be made tedious or punitive by deprivation of toys, books, or of mechanical work. Sewing or knitting should only be allowed when those arts have been already acquired, and new kinds of work should not be attempted. At this period inattention and instability, the two most prominent features of the condition, are to be met, not by correction, but by judicious sympathy. It is advisable that these children should have a light burning in their bedrooms at night, and a grown-up person should sleep in the same room.

The question of exercise must be largely determined by the coexistence of other rheumatic manifestations, as indicated by the temperature, by the state of the heart, and by the presence of pain. In the absence of these there is no harm in letting the child go out for short walks, but it must be remembered that any fright, over-fatigue or excitement may have the result of causing a rapid development of symptoms.

By the adoption of these general hygienic precautions and by feeding the child well and securing sleep by means of a warm bath at night, and by the administration of sedatives, I think it is quite possible to avert an attack of chorea. For the purpose of securing sleep I have found nothing so useful as the administration of trional in 5 grain doses every six hours. It is rarely, however, that we are called on to treat the very early stages of the disease, as they are generally not recognised till the movements are quite definite.

(2) *Symptomatic.*—The symptomatic treatment of chorea will in the main resolve itself into the treatment of the movements, insomnia, the emotional upsets, the wasting, and of the paralytic phenomena which may constitute the great feature of an attack of chorea.

*Movements.*—Directly these are marked the child should be put to bed and kept there. The bed should not be too large and should be protected at the sides, so that there is no risk of the child falling out, and if the movements are at all severe it will be necessary to have the sides of the bed padded. In the worst cases of movement it may be necessary to make the bed up on the floor, but this is a measure which has great disadvantages, as it increases the difficulty of nursing the child, and is not without risks from the liability to draught and dust, while the unusual situation is not particularly reassuring to an excitable child.

When movements are marked, water-beds are not indicated, though in the paralytic form of chorea, and when there is very marked wasting, they are of the greatest use. Although bruises on the shins are so common as to constitute a trade mark of chorea, yet sores in the form of abrasions of the skin with subsequent pus infection are surprisingly rare, and I have never seen a bed-sore develop in hospital, though I have seen cases in which, from bad nursing, sores had developed previous to admission. One of the worst I ever saw was in a boy who developed the paralytic form of chorea and pericarditis, and who had had a blister applied all down his spine previous to his admission into hospital. When the movements distress a child much, considerable relief can be obtained by wrapping the child up in a blanket and then tucking the child firmly into bed. It is important in such cases to avoid anything which would seem to the child to savor of punishment. Choreic movements should not be allowed to go on unrestrained. The measures we may employ to diminish the movements may be divided into (i.) external applications and (ii.) internal medication.

(i.) *External Applications.*—These include packs, baths, douches, sponging, massage and electricity. Of all the external agents there is none so satisfactory as the use of the hot-pack. The child is wrapped in a blanket, which is wrung out in hot water, covered over with a mackintosh sheet, and then with another dry blanket, and thus enveloped, is tucked up in bed. In such a warm pack a child may be left for several hours. In a case recently under my care, where, in spite of the internal administration of sedatives, the movements were becoming more marked and the insomnia increasing, the application of the hot-pack was followed by ten hours continuous sleep with a very marked diminution of the movements. This form of treatment is nearly always grateful to the patient.

Cold-packs are indicated when the case is complicated with severe pyrexia, but apart from that they do not offer any advantages over the hot-packs.

Cold douches and tepid sponging find their chief utility during the period of convalescence or in very chronic cases.

Blisters and counter-irritation along the spine need only to be mentioned to be condemned.

Massage is undoubtedly of great benefit when the movements are subsiding, but has its greatest utility in the treatment of the wasting and of the loss of power after prolonged chorea, either of the motor or the paralytic variety.

Electrical treatment may be useful under similar circumstances, but it has seemed to me to offer no advantages and to present many disadvantages compared with the treatment by means of simple massage.

During convalescence the coördination of the muscles may be increased by practice, such as may be obtained by playing with marbles on a solitaire board or by various kindergarten occupations; but nothing must be attempted which, if failed in, would give the child occasion for reproof or regret.

(ii.) *Internal Medication.*—The length of the list of drugs which have been recommended in the treatment of chorea is a sufficient commentary on their efficacy in the disease. For many years arsenic has been perhaps the most widely employed drug. Originally given in moderate doses, it has of late years been pushed so as to bring patients rapidly and markedly under its influence. In some cases this line of treatment has been followed by distinct improvement, but in other cases it has failed and the therapeutical problem has been complicated by the necessity of treating a case of chorea and one of arsenical poisoning in the same individual. At the present time I have quite given up the use of arsenic in the acute stages of chorea, though it is useful in combination with iron and nux vomica in the treatment of convalescence and in the paralytic forms. Ergot has also been recommended, but in my hands has not justified the claims made for it. In combination with strychnine I have also failed to get good results from its use.

(3) *"Specific"* treatment of chorea has been based on the view, which I hold very firmly, that in the vast majority of cases chorea is a rheumatic manifestation. Accepting this as the correct pathology of the disease, treatment has been directed to combat the rheumatic poison which we assume to be present in the rheumatic state. First and foremost among these remedies we place the salicylates and aspirin.

My experience of the treatment of chorea with salicylates have been far less satisfactory than that of others who have advocated the use of the drug, for though I have rarely seen any definite improvement follow its use I have on several occasions seen chorea develop in children who were actually under the influence of salicylates for the treatment of some active rheumatic condition. When there are active rheumatic conditions such as arthritis, tonsillitis, pyrexia and possibly, in some cases, recent peri- or endocarditis, then salicylates are useful for the treatment of these conditions, though I have not been able to convince myself that they produce any definitely beneficial effect on the chorea. Dr. Lees, however, speaks very decidedly in favor of the administration of large doses (400 grains to 500 grains per diem) of salicylate of soda, but my experience with less heroic doses has not been so favorable as far as the chorea is concerned, while the risks of the production of conditions of acidosis must be prominently borne in mind. In the present state of our knowledge of rheumatism I think we are not justified in taking up the position that whatever is rheumatic must therefore respond to salicylates. One striking example in illustration of this is to be seen in the case of rheumatic nodules, which few would deny to be, in children, pathognomonic of rheumatism, and yet these nodules do not show any definite response to treatment by the salicylates. I have, in cases of chorea uncomplicated with other rheumatic manifestations, discarded the salicylate treatment.

In uncomplicated chorea the best results I have seen have followed on the administration of sedatives. For this purpose bromides and chloral have been extensively employed, but though useful in some cases have been very unsatisfactory in many, and chloral is not a drug which should be used without great care, especially in a disease in which the heart so often is affected, either with dilatation or with organic valvular mischief. More than two years ago, following Dr. Essex Wynter's suggestion, I used chloretone as a sedative, and had very gratifying results from its use; the period of attack, the severity of the movements and the mental instability were all favourably influenced by the drug, but it has some minor disadvantages in this, that it is rather apt to make the children too drowsy, and there is sometimes produced an erythematous rash and the eyes get a puffy appearance not unlike that produced by whooping-cough, but unaccompanied by albuminuria. While chloretone has proved of distinct use in the treatment of chorea, I have had distinctly better results from the use of trional. Under its influence the movements subside more rapidly than with any other drug I have employed, the mental condition is distinctly improved, there is less instability, and this is not accompanied by as much drowsiness or heaviness as I have observed with chloretone. I have recently tried a new drug called bromural which is *a* monobrom-isovalerianylurea. Although this drug has proved very useful in the treatment of the restlessness and sleeplessness which accompany so many cases of heart disease, yet its influence on the progress of those cases of chorea on which I have tried it has not been more beneficial than that of either chloretone or of trional.

In trional we possess a drug which has a very distinctly beneficial effect in chorea, both in alleviating the symptoms and, what is also a very important point, in reducing the time required for the treatment of the disease. Trional has not in my experience ever caused any cardiac depression, and the only unfavorable condition I have observed in cases taking it has been that some of the children have had rather vivid dreams, but this is only exceptionally met with. I have never seen any delirium following its use.

Trional is practically insoluble in water, so that it is best administered in the form of a cachet or else in "tabloid" form, or given in suspension. Given in these forms the drug does not cause gastric disturbance, vomiting is not observed, nor does the appetite suffer. The following mode of exhibiting trional has been suggested:

| | | |
|---|---|---|
| ℞ | Trional ........................................ | gr. xv. |
| | P. sacc. alb. ........................................ | ʒii. |
| | Gum. trag. ........................................ | gr. iii. |
| | Misce. Fiat. emuls. | |
| | Gum. arab ........................................ | gr. iii. |
| | Aq. flor. aurant. ........................................ | ʒii.ss. |
| | Aq. laur. ceras. ........................................ | ʒss. |

One-third part to be taken in milk or water as a single dose.

The dose of trional with which I generally commence is 5 grains three times a day for a child over 4, but this dose should be increased by more frequent administration, so that the child is taking 5 grains every six or every four hours. It is, I believe, more advantageous to administer the smaller doses at shorter intervals than to give larger doses at longer intervals.

There is, at the present time, no specific for the treatment of chorea; certainly there are cases in which even trional fails to arrest or even to diminish to an appreciable extent the movements in chorea. Such cases, I am sure, are best treated by hot-packs if the movements are severe or if the sleeplessness is marked. In the severest forms it may be necessary to resort to the inhalation of chloroform vapor, but this is very rarely called for.

When choreic movements are subsiding the dose of trional should be diminished by reducing the frequency of administration, and during convalescence preparations of iron, arsenic, and nux vomica, or cod-liver oil and iron, will prove most useful. Headache, which is a not infrequent accompaniment of chorea, is best treated by aspirin or by a combination of phenacetin and citrate of caffein. For maniacal conditions the hot-pack is of the greatest use, while in hyperpyrexia the use of the cold pack or cold bath is imperative.

When we meet with concurrent chorea and arthritis, tonsillitis, pyrexia, or peri-, myo or endocarditis, then it is best to administer both trional and an anti-rheumatic drug, and for this purpose in children I prefer aspirin or salol to the salicylate of soda. In cases of pericarditis, the application of an ointment containing 1 dram of oil of gaultheria to 1 ounce of lanolin over the præcordium will often give great relief.

All through the course of an attack of chorea attention must be paid to the diet, which must be liberal, easily digested and, in the paralytic forms of the disease, administered at short intervals and in a readily assimilable form. Alcohol, which is very rarely called for in ordinary cases of chorea, is very useful in cases marked by much wasting, and in the paralytic forms of the disease; it is best given in the form of port wine.

In the paralytic forms of the disease food and sleep are the chief desiderata. Neither the anti-rheumatic or the sedative drugs have seemed to me to exercise any beneficial effects, but strychnine, arsenic, iron, and cod-liver oil, combined with careful feeding and with massage, have given the best results.

It is sometimes a difficult matter to determine at what period a choreic child should be allowed to get up. In the absence of complications, provided the heart is not dilated and that the knee-jerks are present, we may with benefit let a child get up as soon as it can feed itself quite well and can walk without assistance. It is at the stage of convalescence that a stay in the country is most productive of benefit.

